# Recurrence after skin-sparing mastectomy and immediate transverse rectus abdominis musculocutaneous flap reconstruction for invasive breast cancer

**DOI:** 10.1186/1477-7819-11-194

**Published:** 2013-08-14

**Authors:** Tsung-Jung Liang, Being-Whey Wang, Shiuh-Inn Liu, Ming-Hsin Yeh, Yu-Chia Chen, Jin-Shyr Chen, King-Tong Mok, Hong-Tai Chang

**Affiliations:** 1 Division of General Surgery, Department of Surgery, Kaohsiung Veterans General Hospital, 386 Ta-Chung 1st Road, Kaohsiung 813, Taiwan; 2 National Yang-Ming University School of Medicine, NO. 155, Sec. 2, Linong Street, Taipei, Taiwan; 3 Division of Plastic Surgery, Department of Surgery, Kaohsiung Veterans General Hospital, 386 Ta-Chung 1st Road, Kaohsiung 813, Taiwan

**Keywords:** Invasive breast cancer, Skin-sparing mastectomy, Immediate breast reconstruction, Transverse rectus abdominis musculocutaneous flap, Recurrence

## Abstract

**Background:**

The aim of this study was to evaluate the recurrence pattern after skin-sparing mastectomy (SSM) and immediate breast reconstruction (IBR) using transverse rectus abdominis musculocutaneous (TRAM) flap in patients with invasive breast cancer.

**Methods:**

From 1995 to 2010, patients with invasive breast cancer who underwent SSM followed by IBR using TRAM flap were retrospectively reviewed. The pattern of the first recurrence event was recorded.

**Results:**

We identified 249 consecutive patients with invasive breast cancer, two-thirds of whom (67.1%) were diagnosed with stage II or stage III disease. During a median follow-up period of 53 months, three (1.2%) local, 13 (5.2%) regional, 34 (13.7%) distant, and five (2.0%) concurrent locoregional and distant recurrences were observed. The median time to recurrences was 26 months (range, 2 to 70 months) for all recurrences, 23 months (range, 2 to 64 months) for locoregional recurrences, and 26 months (range, 8 to 70 months) for distant recurrences. All local recurrent lesions were detectable by careful physical examination, and detection of local recurrence suggested the presence of distant metastasis (60.0%). In contrast to distant metastasis, the risk of locoregional recurrence did not increase significantly with an increase in disease stage. The 5-year overall, locoregional relapse-free, and distant relapse-free survival rates were 89.7%, 90.8%, and 81.6%, respectively.

**Conclusions:**

SSM followed by immediate reconstruction using TRAM flap is an oncologically safe procedure even in patients with advanced-stage disease. Detection of local recurrence is crucial and can be aided by a thorough physical examination.

## Background

Skin-sparing mastectomy (SSM) followed by immediate breast reconstruction (IBR) has become a common and widely used procedure for patients with ductal carcinoma *in situ* and early-stage breast cancer [1]. The oncological safety and superior cosmetic outcomes of this approach have been demonstrated in several studies [2,3]. The use of this approach is still debated, however, and the implementation of SSM for advanced-stage disease has not been thoroughly investigated [4-6].

One of the major concerns regarding SSM and IBR is local recurrence [7,8]. Inadequate skin excision after SSM may increase the theoretical risk of recurrence. Another concern is that IBR may obscure a recurrent lesion, thereby delaying its diagnosis and subsequent management [9,10]. Furthermore, no clear consensus has been established regarding the ideal screening tool for reconstructed breast tissue [9]. Unsurprisingly, therefore, some authors have speculated that the combination of SSM and IBR may result in inferior oncological outcomes, especially in patients with more advanced disease stages [11].

Among the various types of breast reconstruction, use of the transverse rectus abdominis musculocutaneous (TRAM) flap is a popular choice for either immediate or delayed reconstruction [10]. The flap transfers sufficient tissue to create a breast with excellent bulk and simultaneously provides an abdominoplasty. Use of the TRAM flap is thus the most common reconstruction method applied in our institution. Most published studies that investigated SSM and IBR enrolled patients using some degree of selection (either early-stage patients or advanced-stage patients were included), evaluated a small number of cases, and employed more than one type of reconstruction method [1,11,12]. The patients in the present study were relatively unselected and received only one type of reconstruction.

The aim of this study was to evaluate the incidence and pattern of recurrence in a broad spectrum of breast cancer patients regardless of the tumor stage after treatment with SSM and IBR with TRAM flap.

## Methods

### Patients

Between August 1995 and December 2010, data on all breast cancer patients who underwent SSM and IBR using TRAM flap at Kaohsiung Veterans General Hospital were retrospectively reviewed. In total, 335 patients were treated during this period. Patients with local recurrence following previous lumpectomy or mastectomy for prophylaxis and those with a follow-up period < 2 years were excluded (*n* = 38). Patients with purely *in situ* disease were also excluded (*n* = 48). A total of 249 patients with invasive breast cancer were enrolled in this study. Diagnoses of all patients were pathologically confirmed, and the disease was staged according to the seventh edition of the American Joint Committee on Cancer staging system [13]. Inform consent was obtained from all patients before operation. This study was approved by the institutional review board in Kaohsiung Veterans General Hospital.

### Surgical treatment

SSM was defined as removal of the whole breast tissue, the nipple–areola complex, the biopsy scar, and the skin overlying superficial tumors. The remaining native skin envelope and inframammary fold were preserved. Axillary lymph node dissection was subsequently performed via an axillary incision. All IBR procedures were performed using a pedicled TRAM flap. Patients were all counseled preoperatively regarding the types of mastectomy and reconstruction options. Risks and benefits of different methods were thoroughly explained.

### Neoadjuvant and adjuvant treatment

Neoadjuvant chemotherapy with an anthracycline-based regimen was considered for patients presenting with locally advanced disease. The number of neoadjuvant chemotherapy cycles depended upon individual clinical response and the surgeon’s discretion. Adjuvant chemotherapy was indicated for patients with positive lymph node status. Among patients without lymph node involvement, chemotherapy was considered for those with unfavorable prognostic features such as negative estrogen receptor status, HER-2 overexpression, high histologic grade, presence of lymphovascular invasion, and large tumor size. Treatment was commenced once patients recovered from surgery. Anthracycline-based regimens alone or anthracycline-based regimens followed by taxane-based regimens were the two most common protocols.

In all cases, radiation was delivered postoperatively and after the completion of adjuvant chemotherapy. The general indications for postmastectomy radiotherapy included skin involvement, positive lymph node status, and tumor size >5 cm. The radiation field included the ipsilateral chest wall, the internal mammary chain, and the supraclavicular fossa. Patients with positive hormone receptor status received appropriate hormone therapy, usually tamoxifen after surgery or completion of adjuvant chemotherapy.

### Follow-up and outcome

The follow-up period was calculated from the date of SSM to the date of last patient contact. Local recurrence was defined as a biopsy-proven recurrent tumor within the ipsilateral anterior chest wall, including the skin, subcutaneous tissue, and muscle. Regional recurrence was defined as tumor involvement of ipsilateral axillary, internal mammary, supraclavicular, or infraclavicular lymph nodes. Recurrence at all other locations was classified as distant metastases.

Patients were regularly followed-up every 3 to 6 months for the first 5 years and every 12 months thereafter. Reconstructed breasts were routinely checked by physical examination and sonography. Annual mammography was performed on the contralateral healthy breast. Abnormal clinical findings might be evaluated by further studies, including chest radiographs, sonography for liver, bone scan, and tumor markers (CA-153 and carcinoembryonic antigen).

### Statistical analyses

Disease-free survival and overall survival were estimated using the Kaplan–Meier method, and differences were compared using the log-rank test. Factors that possibly related to locoregional recurrence, including age, histologic grade, tumor size, lymph node status, American Joint Committee on Cancer stage, and hormone receptor status, were analyzed by univariate comparison. Subsequent multivariate analysis was not performed because of the small sample size. All analyses were performed using Statistical Program for Social Sciences (SPSS 18.0 for Windows; SPSS, Inc., Chicago, IL, USA). *P* < 0.05 was considered significant.

## Results

### Patient characteristics

The characteristics of the 249 patients are presented in Table 1. The median age at the time of diagnosis was 41 years (range, 22 to 62 years). All patients had invasive breast cancer, and patients with stage II and stage III disease accounted for two-thirds (67.1%) of the study population (Table 2).

**Table 1:** Patient characteristics

**Variable**	**Data**
Age (years)	41 (22 to 62)
Laterality	
Left	132 (53.0)
Right	117 (47.0)
Histological type	
Ductal	228 (91.6)
Lobular	11 (4.4)
Mucinous	5 (2.0)
Medullary	5 (2.0)
Grade	
1	32 (12.9)
2	132 (53.0)
3	85 (34.1)
Tumor size (cm)	2.2 (0.1 to 11)
T stage	
T1	110 (44.2)
T2	130 (52.2)
T3	6 (2.4)
T4	3 (1.2)
Lymph node status	
N0	157 (63.1)
N1	59 (23.7)
N2	25 (10.0)
N3	8 (3.2)
Estrogen receptor	
Positive	162 (65.1)
Negative	87 (34.9)
Progesterone receptor	
Positive	137 (55.0)
Negative	112 (45.0)

Data presented as median (range) or *n* (%).

**Table 2:** Tumor staging

**Disease stage**	***n***	**%**
I	82	32.9
IIA	89	35.7
IIB	41	16.5
IIIA	26	10.4
IIIB	4	1.6
IIIC	7	2.8

### Treatment characteristics

Sixteen patients (6.4%) received neoadjuvant chemotherapy with two to five cycles of 5-fluorouracil, anthracycline, and cyclophosphamide (median, four cycles). One hundred and ninety-six patients (78.7%) were treated with adjuvant chemotherapy using anthracycline-based and/or taxane-based regimens after surgery. The median time from surgery to adjuvant chemotherapy was 31 days (range, 14 to 100 days). In only eight patients did this time interval exceed 60 days. Additional adjuvant radiotherapy was administered to 32 patients (12.9%) after the completion of adjuvant chemotherapy. The indication for radiation was lymph node metastasis and/or large tumor size. One-half of the patients (50.6%) received hormone therapy. Tamoxifen was administered to all these patients, except for seven premenopausal patients who received gonadotropin-releasing hormone analogues.

### Recurrence

The median follow-up period was 53 months (range, 24 to 181 months). During the follow-up period, 55 patients (22.1%) experienced recurrence. The patterns of first recurrence are summarized in Table 3. The median time to recurrence was 26 months (range, 2 to 70 months) for all recurrences, 23 months (range, 2 to 64 months) for locoregional recurrences, and 26 months (range, 8 to 70 months) for distant recurrences. Among the 16 patients with locoregional recurrence, the first recurrence event was isolated local recurrence in three patients and regional recurrence alone in the other 13 patients.

**Table 3:** Pattern of first breast cancer recurrence stratified by stage

				** Recurrence**		
**Stage**	***n***	**Local**	**Regional**	**Distant**	**LR + D**	**Overall *n *(%)**
I	82	0	4	4	2	10 (12.2)
II	130	1	9	17	1	28 (21.5)
III	37	2	0	13	2	17 (45.9)
Total, *n* (%)	249	3 (1.2)	13 (5.2)	34 (13.7)	5 (2.0)	55 (22.1)

*n*, patient number; LR + D, concurrent locoregional and distant relapse.

Of the three patients with isolated local recurrence, all recurrent lesions were detectable by palpation, despite the small tumor size (0.8 to 2.2 cm in diameter). Diagnosis was established by either needle cytology or excision biopsy. Wide excision was subsequently performed followed by radiotherapy and chemotherapy. Primary skin closure could be accomplished in all cases without the use of an additional flap or graft. TRAM flap removal was not required during the resection of these recurrent lesions. No further recurrence was detected in two patients. However, the other patient experienced multiple liver and bone metastases 26 months after the first relapse. The 13 patients with regional lymph node recurrence alone underwent lymph node dissection followed by additional chemotherapy and/or radiotherapy. No further recurrence occurred in 10 patients. In contrast, three patients subsequently developed another recurrence 8, 10, and 56 months after the first relapse. In total, all but one patient (15/16, 93.8%) with locoregional recurrence as the first recurrence event were alive at the end of the follow-up period.

**Figure 1:**
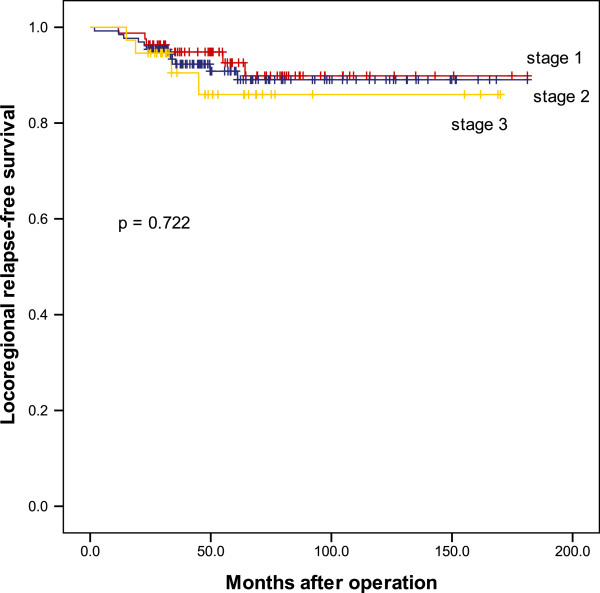
Kaplan–Meier curves for locoregional relapse.

**Figure 2:**
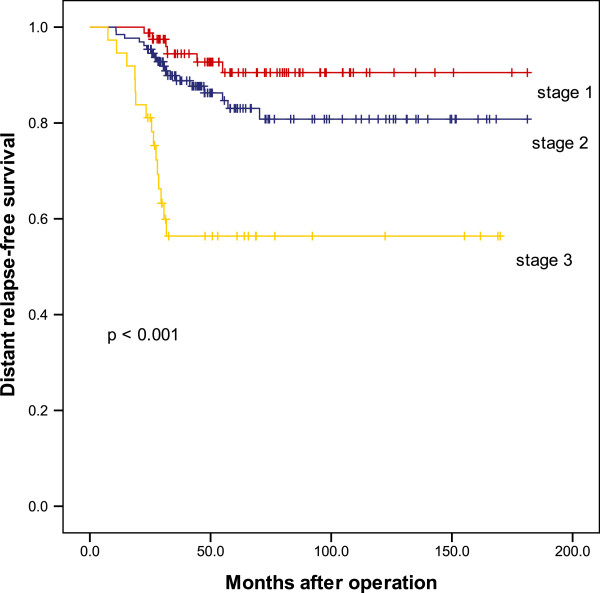
Kaplan–Meier curves for distant relapse.

Concurrent locoregional and distant recurrence occurred in five patients (2.0%): three patients showed regional and distant recurrence and two patients showed local, regional, and distant recurrences. Local recurrences that occurred in this group of patients presented as palpable nodules or erythematous skin. Despite multidisciplinary treatments, all five patients died, with a median survival of 13 months after recurrence (range, 5 to −46 months). On stratification according to tumor stage, distant recurrence was significantly more frequent in patients with advanced-stage disease (*P* <0.001), whereas no such correlation was observed for locoregional recurrence (*P* = 0.722) (Figures 1 and 2). In univariate analysis, factors associated with locoregional recurrence after SSM and IBR were tumor size > 5 cm (*P* = 0.042) and negative progesterone receptor status (*P* = 0.004).

Twenty-six patients (10.4%) died during the follow-up period, and all deaths were due to progression of metastatic disease. Distant recurrence was the first recurrent event in all of these patients except for one, in whom ipsilateral supraclavicular lymph node involvement alone was the first recurrence event. The average time to death after first recurrence was 25 months (range, 1 to 75 months), and 16 patients (61.5%) died within 2 years. The 5-year overall, locoregional relapse-free, and distant relapse-free survival rates were 89.7%, 90.8%, and 81.6%, respectively.

## Discussion

This study included 249 consecutive patients with invasive breast cancer who underwent SSM and IBR using pedicled TRAM flap. The avoidance of patient selection and the use of only one type of reconstruction method and a standardized management protocol may provide a more thorough perspective on this treatment approach.

One concern regarding immediate reconstruction is whether it hinders or delays the detection of local recurrence [14]. All five local recurrences (three local alone and two concurrent local and systemic) that developed in our series were detectable by physical examination even in cases of recurrent tumor size <1 cm in diameter. This result is consistent with two other series in which all three and five of six local recurrences, respectively, were detectable by physical examination [9,15]. Of 16 local recurrences after immediate TRAM flap reconstruction reported by Howard and colleagues [10], all were identified by physical examination or subjective symptoms that were subsequently confirmed by physical examination or imaging studies. Our experience also highlights the importance of physical examination, because the presence of local recurrence may herald systemic metastasis either synchronously or metachronously. Three patients with local recurrences (3/5, 60.0%) developed distant metastasis in our series.

In addition to physical examination, we routinely use sonography for screening the reconstructed breast. The ideal modalities for recurrence surveillance in this population have not yet been determined. Some authors recommended routine mammography and have used it as an aid in the differentiation between fat necrosis and cancer. However, these two different lesions cannot always be distinguished solely on the basis of mammographic findings [9,16]. Pathological diagnosis remains the gold standard, and biopsy is warranted for any new or persistent lesion in the reconstructed breast.

The reported local recurrence rates after SSM and IBR in studies with more than 100 patients range from 0.2 to 7.0% [17,18]. When limiting the study population to patients with locally advanced breast cancer undergoing the same procedure, the reported rates of local recurrence are between 1 and 10% [6,11,19]. The local recurrence rate for stage III patients (2/37, 5.5%) in our study is comparable with that reported previously [6,11,19]. Despite the relatively small number of patients with stage III disease in this series, our findings might strengthen the rational use of SSM and immediate TRAM flap reconstruction in patients with advanced-stage disease. A larger study with more patients is warranted to further address this issue. Several studies have reported no significant difference in recurrence pattern or incidence between patients undergoing SSM and conventional mastectomy [17,20]. Comparable results have also been observed for patients with stage III disease [17].

The median time to locoregional recurrence was 23 months, 3 months shorter than that to distant relapse, in our study. This finding is comparable with previously reported results [10,21]. In a series of 1,392 breast cancer patients who underwent mastectomy, Crowe and colleagues found that locoregional recurrence occurred within the first 3 years in most cases, with a peak in the second year [22]. The recurrence rate then dropped sharply and remained relatively constant over a long period of time. However, recurrence occurring 10 years after mastectomy has been reported, and a longer follow-up time is warranted [21].

The rate of distant recurrence was significantly higher in patients with advanced-stage disease, and distant metastasis was the cause of all deaths that occurred in our series. Despite multimodal treatment, > 60% of patients died within 2 years after first distant relapse. In contrast to the distant metastasis rate, the locoregional recurrence rate did not increase significantly from stage I disease to stage III disease. This difference may be ascribed to the aggressive use of systemic therapy in this study.

In recent years, a trend of decreased local recurrence rate has been observed [17]. Some authors considered this decline attributable, at least in part, to the increasing use of adjuvant therapy [17]. Our result may further support the effectiveness of systemic treatment in reducing the risk of locoregional recurrence and narrowing the gap in outcome between different stages. Nevertheless, such intensive adjuvant therapy can pose a heavy financial burden to patients. In Taiwan, most of the cancer treatment cost is covered by national health insurance. Thus, this type of treatment was affordable for the majority of patients in this series.

Based on our results, solitary local or regional recurrence as the first recurrence event was associated with a better prognosis than the development of locoregional recurrence as part of systemic metastasis. Differentiation between different recurrence patterns may thus be of great help in predicting prognosis, and a thorough metastatic survey at the time of the first recurrence is warranted. Recently, the use of nipple-sparing mastectomy has been on the rise, although its clinical application is usually limited to prophylactic risk reduction and ductal carcinoma *in situ*[23]. The oncologic safety of this procedure remains the major concern that hampers its application. Our results may further support the possible implementation of nipple-sparing mastectomy for cases of advanced disease.

## Conclusion

Our study showed that SSM followed by IBR using TRAM flap was an oncologically safe procedure. The locoregional recurrence rate was acceptable even in cases of advanced-stage disease. The TRAM flap did not hinder the detection of recurrence. The first course of postoperative chemotherapy could be delivered without delay. In conjunction with adjuvant treatment, this procedure can be applicable to advanced-stage disease.

## Abbreviations

IBR: Immediate breast reconstruction; SSM: Skin-sparing mastectomy; TRAM: Transverse rectus abdominis musculocutaneous.

## Competing interests

The authors declare that they have no competing interests.

## Authors’ contributions

TJ-L, B-WW, and H-TC participated in the design of the study and drafted the manuscript. M-HY, Y-CC, and J-SC participated in data acquisition an analysis. S-IL and K-TM carried out the literature review and revised the manuscript. All authors read and approved the final manuscript.
